# Lipid accumulation product, visceral adiposity index and risk of chronic kidney disease

**DOI:** 10.1186/s12882-022-03026-9

**Published:** 2022-12-15

**Authors:** Alexander L. Bullen, Ronit Katz, Ujjala Kumar, Orlando M. Gutierrez, Mark J. Sarnak, Holly J. Kramer, Michael G. Shlipak, Joachim H. Ix, Suzanne E. Judd, Mary Cushman, Pranav S. Garimella

**Affiliations:** 1 Nephrology Section, Veterans Affairs San Diego Healthcare System, La Jolla, CA USA; 2 Division of Nephrology & Hypertension, University of California San Diego, 200 W Arbor Dr.. M/C 8409 MPF L030, San Diego, CA 92103 USA; 3 Division of Obstetrics and Gynecology, University of Washington, Seattle, WA USA; 4 Division of Nephrology, UAB Heersink School of Medicine, Birmingham, AL USA; 5 Division of Nephrology, Tufts Medical Center, Boston, MA USA; 6 Division of Nephrology and Hypertension, Loyola University Medical Center, Maywood, IL USA; 7 Kidney Health Research Collaborative, Department of Medicine, University of California, San Francisco, CA USA; 8 Department of Medicine, San Francisco VA Medical Center, San Francisco, CA USA; 9 Department of Biostatistics, UAB School of Public Health, Birmingham, AL USA; 10 Division of Hematology and Oncology, University of Vermont, Colchester, VT USA

**Keywords:** Obesity markers, Chronic kidney disease, Albuminuria, Kidney failure

## Abstract

**Background:**

Lipid accumulation product (LAP) and visceral adiposity index (VAI) are novel, non-imaging markers of visceral adiposity that are calculated by using body mass index (BMI), waist circumference (WC) and serum lipid concentrations. We hypothesized that LAP and VAI are more strongly associated with adverse kidney outcomes than BMI and WC.

**Methods:**

Using data from the REasons for Geographic and Racial Differences in Stroke (REGARDS) study, we used multivariable logistic regression to evaluate associations of LAP, VAI, BMI and WC with incident chronic kidney disease (CKD), (incident eGFR < 60 ml/min/1.73m^2^ and > 25% decline).

**Results:**

Among the overall cohort of 27,550 participants, the mean baseline age was 65 years; 54% were women; and 41% were African American. After a median of 9.4 years (IQR 8.6, 9.9) of follow-up, a total of 1127 cases of incident CKD were observed. Each two-fold higher value of VAI (OR 1.12, 95% CI 1.04, 1.20), LAP (OR 1.21, 95% CI 1.13, 1.29), WC (OR 2.10, 95% CI 1.60, 2.76) and BMI (OR: 2.66, 95% CI 1.88, 3.77), was associated with greater odds of incident CKD.

**Conclusions:**

LAP and VAI as measures of visceral adiposity are associated with higher odds of incident CKD but may not provide information beyond WC and BMI.

**Supplementary Information:**

The online version contains supplementary material available at 10.1186/s12882-022-03026-9.

## Introduction

Obesity is associated with incident hypertension, diabetes, cardiovascular disease (CVD), and kidney disease [1, 2]. The prevalence of chronic kidney disease (CKD) has increased in parallel with obesity in the past three decades [3]. Obesity can increase the risk of incident CKD and progression of CKD by promoting hypertension and diabetes mellitus (DM), but may also directly lead to loss of kidney function due to secondary focal segmental glomerulosclerosis [4, 5]. While body mass index (BMI) is the most common measure of body size used to diagnose and stratify obesity, BMI includes both fat and muscle mass and consequently may be an imprecise measure of obesity [6]. Furthermore, patients with CKD often have low lean muscle mass and volume overloaded thus reducing the utility of BMI [7].

Central obesity has been proposed as an alternate measure of obesity and can further be divided into the subcutaneous and visceral adiposity; the latter has been shown to be particularly strongly associated with CVD risk factors [8–11], reduced estimated glomerular filtration rate (eGFR), microalbuminuria, CKD, and mortality [12–15]. Waist circumference (WC), and waist-to-hip ratio are accepted measures of central obesity [16] but are not well correlated with visceral adiposity quantified by imaging [17–19]. Although imaging modalities such as computed tomography (CT), magnetic resonance imaging (MRI), or dual energy X-ray absorptiometry (DEXA) can differentiate subcutaneous and visceral adiposity accurately [20, 21], these techniques are expensive, time-consuming, and associated with radiation exposure.

Recently, two metrics which may better estimate visceral adiposity without imaging have been proposed. The first, lipid accumulation product (LAP), is an index calculated using WC and fasting triglycerides (TG). The lipid accumulation product by adding fasting concentration of circulating triglycerides to waist circumference seeks to express the index of the anatomic and physiological changes associated with visceral fat deposition [22] over the less specific overaccumulation of weight. The second is the visceral adiposity index which follows the same premise, but it additionally adds the BMI and high-density lipoprotein cholesterol (HDL). VAI has been shown to be a good indicator of visceral adipose tissue measured with MRI [23]. BMI lacks discriminatory power between fat and lean tissues, which leads to marked variation in metabolic disease among individuals with similar levels of adiposity as determined by BMI [24]. Conversely, VAI and LAP have identified individuals with cardiovascular risk factors such as insulin resistance, impaired glucose tolerance, and hypertension with normal BMIs [25]. One cross sectional study suggested that both VAI and LAP were more strongly associated with CKD than BMI and WC in women [26]. To date, no studies have evaluated the association of these novel measures of visceral obesity with longitudinal decline in kidney function. We hypothesized that visceral adiposity as measured by LAP and VAI would be more strongly associated with incident CKD, eGFR decline, and incident kidney failure than BMI and WC after adjusting for confounders.

## Material and methods

### Participants

We used data from the REasons for Geographic and Racial Differences in Stroke (REGARDS) study, a population-based prospective cohort of community-dwelling individuals aged 45 years and older. The study was designed to evaluate the risk factors that contribute to disproportionate mortality linked with stroke in the Southeast United States and among African Americans. A total of 30,239 adults were recruited between January 2003 and June 2007. Trained staff collected baseline information during a preliminary phone interview followed by an in-home physical examination. The in-home visit included measurement of blood pressure, anthropometric measurements and collection of fasting (10–12 hour) blood and spot urine specimen. Details of the study have been published previously [27]. Between April 2013 and December 2016, participants were invited to undergo a second in-person visit during which the baseline procedures were repeated. Among the 16,150 participants who participated in the second assessment, 15,938 completed the second telephone-administered assessment and 14,449 had in-person assessments. All participants provided written informed consent and Institutional Review Boards of all participating institutions approved the study.

### Exposure variables

BMI was measured as weight (kg)/height (m^2^) and categorized as ≤25.1 (reference group), 25.2–28.3, 28.4–32.5, > 32.5. Waist circumference was measured mid-way between the lowest rib and the iliac crest using a tape measure with the participant standing and was categorized in cm as < 86.3 (reference group), 86.4–95.2, 95.3–105.4, and > 105.4. VAI was calculated as: [WC/39.68 + (1.88 × BMI)] × (TG /1.03) × (1.31/ HDL) for men, VAI = [WC/36.58 + (1.89 × BMI)] × (TG/0.81) × (1.52/HDL) for women [23]. LAP was calculated as: (WC-65) × TG for men, and (WC-58) × TG for women [22]. Serum lipids including total cholesterol (TC), HDL and fasting TG were measured by colorimetric reflectance spectrophotometry.

### Covariates

Coronary heart disease (CHD), stroke, and smoking status were obtained by self-report during the telephone interview. DM was defined as a fasting glucose ≥126 mg/dL, or a non-fasting glucose ≥200 mg/dL, or self-reported use of antidiabetic medications. Hypertension was defined as either self-reported use of antihypertensive medications or a systolic blood pressure ≥ 140 mmHg or a diastolic blood pressure ≥ 90 mmHg measured during the home examination at baseline visit. Serum creatinine was calibrated to an international isotope dilution mass spectroscopic (IDMS)-traceable standard and was measured by colorimetric reflectance spectrophotometry (Ortho Vitros Clinical Chemistry System 950IRC, Johnson & Johnson Clinical Diagnostics, www.orthochemical.com). Estimated glomerular filtration rate (eGFR) was calculated using the combined CKD-EPI creatinine and cystatin C equation [28]. Urine albumin and creatinine were measured in a random spot specimen by nephelometry (BN ProSpec Nephelometer, Dade Behring, Marburg, Germany) and Modular-P chemistry analyzer (Roche/Hitachi, Indianapolis, IN), respectively.

### Primary and secondary outcomes

The primary outcome was 1) incident CKD, defined as eGFR < 60 ml/min per 1.73 m^2^ and at least 25% decline in individuals with baseline eGFR > 60 ml/min per 1.73 m^2^ between the baseline and second in-home visit. The secondary outcomes were: 1) progressive eGFR decline, defined as > 30% decrease in eGFR between the baseline and second in-home visit. Both these endpoints required participants to return and provide blood at the second visit, thus we also evaluated incident kidney failure defined as initiation of dialysis as ascertained by United States Renal Data System (USRDS) linkage up to June 2014. This endpoint relied on administrative linkage of baseline data to USRDS, such that all participants provided time at risk for the kidney failure endpoint.

**Fig. 1:**
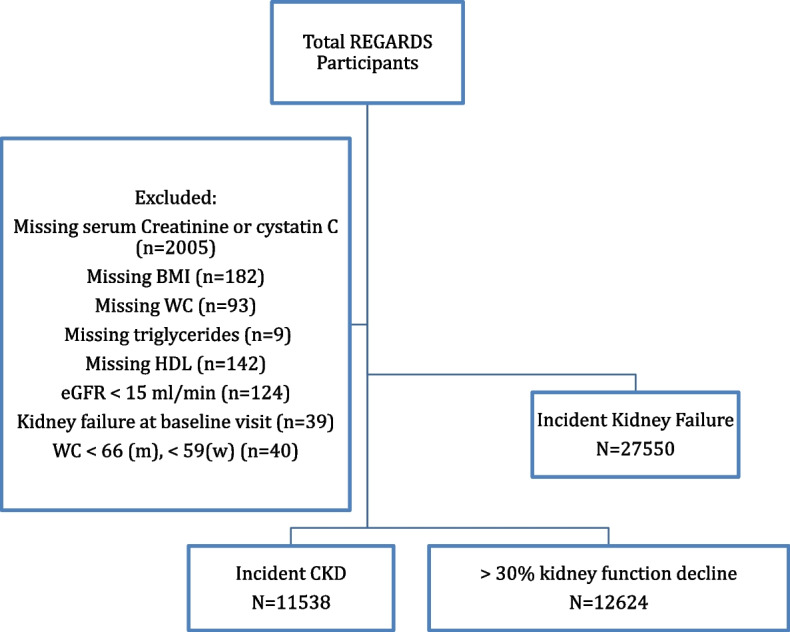
Flowchart of REGARDS Participants for Analysis

For the incident CKD and progressive eGFR analyses, we were limited to participants who had a baseline and follow-up visits and provided blood samples for analysis (Fig. 1). After excluding participants with missing data on serum creatinine or cystatin C, BMI, WC, and fasting triglycerides and/or HDL measurements (*n* = 2430), who had an eGFR < 15 ml/min/1.73 m^2^ or had kidney failure receiving maintenance dialysis (*n* = 163), or who had a waist circumference less than 66 cm (for men) and less than 59 cm (for women) in order to avoid having negative values for LAP and VAI (*n* = 40), a total of 11,538 participants were available for analyses of incident CKD cohort and 12,624 participants were available for analyses of progressive eGFR decline. All individuals who did not meet any exclusion criteria and were successfully linked to USRDS data were included in analyses of incident kidney failure (*N* = 27,550) (Fig. 1).

### Statistical analysis

Univariate parameters were calculated as mean ± standard deviation (SD) or counts and proportions across quartiles of LAP and VAI. Logistic regression was used to determine the association of VAP and VAI with incident CKD and progressive eGFR decline. All anthropometric measures (LAP, VAI, BMI and WC) were modeled both as continuous variables and quartiles. We used a series of sequential models to adjust for confounders. Model 1 was adjusted for age, sex, and race. Model 2 included model 1 plus presence of comorbid conditions (CHD, stroke, hypertension, and DM), and smoking status. Finally, model 3 included model 2 plus baseline eGFR and urine albumin to creatinine ratio (UACR). We tested for interaction between the novel markers of obesity and sex and risk for incident CKD.

Cox proportional hazards models were used to determine the independent associations between LAP, VAI, BMI, and WC with risks of kidney failure using a similar modeling strategy as indicated above. Additional sensitivity analysis for the kidney failure outcome were performed stratifying on baseline eGFR categories (> 60, 45–59, and < 45 ml/min/1.73m^2^). Follow-up time was censored at development of kidney failure, death, or date of last follow-up phone contact, whichever occurred first. We also performed analyses treating death as a competing risk.

We used Wald test statistics to assess the relative importance of adiposity measures for the prediction of kidney failure.

We used logistic regression to evaluate the association between LAP, VAI, BMI and WC with risk of incident albuminuria using the sequential models previously mentioned.

Lastly, since triglycerides and HDL are components of VAI and LAP equations, we performed logistic regression to evaluate if the associations between VAI and LAP and the outcomes were driven by WC and BMI, or whether triglycerides and HDL were also associated with kidney outcomes.

A two-sided *p* value of 0.05 was considered statistically significant for all analyses including interaction terms. Statistical analyses were conducted by using IBM SPSS software, Version 26 (IBM Corp. Released 2019).

## Results

### Characteristics of participants by VAI and LAP quartiles

The mean age was 65 years, 54% were women, 21% had DM, mean baseline eGFR was 82 ml/min/1.73m^2^ and the median baseline UACR was 7 mg/g. Baseline participant characteristics stratified by LAP and VAI levels are presented in Tables 1 and 2 respectively. There were significantly fewer African Americans in the top quartiles of LAP and VAI compared to bottom quartiles. Higher quartiles of LAP and VAI had a greater prevalence of CHD, myocardial infarction, stroke, DM, and hypertension. In addition, higher LAP and VAI quartiles had lower eGFR and higher UACR.

**Table 1:** Baseline characteristics of study participants by quartiles of lipid accumulation product (LAP)

Variables	Q1 ≤ 124.89	Q2 124.9–208.07	Q3 208.08–339.57	Q4 ≥ 339.58	Total
N	6838	6943	6933	6836	27,550
Age (SD)	65 (10)	66 (10)	65 (9)	64 (9)	65 (9)
Female (%)	3996 (58)	3697 (53)	3751 (54)	3520 (52)	14,964 (54)
Race					
White (%)	3977 (58)	3813 (55)	3959 (57)	4650 (68)	16,399 (60)
Black (%)	2861 (42)	3130 (45)	2974 (43)	2186 (32)	11,151 (41)
Smoking					
Never (%)	3424 (50)	3159 (46)	3064 (44)	2733 940)	12,380 (45%)
Former (%)	2336 (34)	2851 (41)	2868 (42)	3006 (44)	11,061 (40%)
Current (%)	1048 (15)	912 (13)	968 (14)	1078 (16)	4006 (15%)
**Co-morbidities**					
CHD (SD)	908 (13)	1127 (16)	1266 (18)	1504 (22)	4805 (17)
MI (SD)	669 (10)	771 (11)	883 (13)	1071 (16)	3394 (12)
Stroke (SD)	300 94)	431 (6)	452 (7)	507 (7)	1690 (6)
Diabetes mellitus (SD)	620 (9)	1136 (16)	1572 (23)	2384 (35)	5712 (21)
HTN (SD)	3014 (44)	3963 (57)	4433 (64)	4764 (70)	16,174 (59)
HTN medications (%)	2526 (37)	3436 (50)	3911 (56)	4202 (62)	14,075 (51)
SBP mm Hg (SD)	123 (17)	127 (16)	129 (16)	131 (16)	127 (17)
DBP mm Hg (SD)	74 (9)	76 (9)	77 (10)	78 (10)	77 (10)
eGFR ml/min/1.73m^2^ (SD)	87 (2)	83 (20)	81 (21)	77 (21)	82 (21)
UACR (IQR)	7 [5, 13]	7 [5, 14]	7 [5, 16]	9 [5, 23]	7 [5, 16]
Total cholesterol mg/dL (SD)	187 (37)	189 (39)	192 (39)	200 (44)	192 (40)
LDL cholesterol mg/dL (SD)	110 (32)	115 (34)	116 (35)	114 (38)	114 (35)
HDL cholesterol mg/dL (SD)	62 (17)	54 (15)	49 (14)	43 (12)	52 (16)
Triglycerides mg/dL (IQR)	71 [58, 88]	95 [79, 115]	125 [103, 152]	198 [155, 257]	111 [81, 158]
***Measures of Adiposity***					
VAI (IQR)	1.87 [1.39, 2.48]	2.95 [2.29, 3.83]	4.35 [3.39, 5.64]	7.87 [5.82, 11.2]	3.63 [2.31, 5.90]
BMI (SD)	24.5 (3.9)	28.2 (4.5)	30.8 (5.6)	33.6 (6.3)	29.3 (6.1)
WC, cm (SD)	82 (10)	93 (10)	100 (11)	109 (14)	96 (15)

*Abbreviations*: *CHD* coronary heart disease, *MI* myocardial infarction, *HTN* hypertension, *SBP* systolic blood pressure, *DBP* diastolic blood pressure, *eGFR* estimated glomerular filtration rate, *UACR* urinary albumin to creatinine ratio, *LDL* low-density lipoprotein cholesterol, *HDL* high-density lipoprotein cholesterol, *LAP* lipid accumulation product, *BMI* body mass index, *WC* waist circumference

**Table 2:** Baseline Characteristics of Study Participants by quartiles of visceral adiposity index (VAI)

Variables	Q1 ≤ 2.31	Q2 2.32–3.64	Q3 3.65–5.91	Q4 ≥ 5.92	Total
N	6873	6915	6886	6876	27,550
Age (SD)	65 (10)	65 (10)	65 (9)	65 (9)	65 (9)
Female (%)	3569 (52)	3850 (56)	3751 (55)	3794 (55)	14,964 (54)
Race					
White (%)	3429 (50)	3756 (54)	4185 (61)	5029 (73)	16,399 (60)
Black (%)	3444 (50)	3159 (46)	2701 (39)	1847 (27)	11,151 (41)
Smoking					
Never (%)	3360 (49)	3188 (46)	3031 (44)	2801 (41)	12,380 (45)
Former (%)	2687 (39)	2761 (40)	2806 (41)	2807 (41)	11,061 (40)
Current (%)	791 (12)	939 (14)	1024 (15)	1252 (18)	4006 (15)
**Co-morbidities**					
CHD (%)	934 (14)	1127 (16)	1249 (18)	1495 (22)	4805 (17)
MI (%)	685 (10)	789 (11)	867 (13)	1053 (15)	3394 (12)
Stroke (%)	308 (5)	412 (6)	461 (7)	509 (7)	1690 (6)
Diabetes mellitus (%)	904 (13)	1184 (17)	1525 (22)	2099 (31)	5712 (21)
HTN (%)	3500 (51)	3971 (57)	4222 (61)	4481 (65)	16,174 (59)
HTN medications (%)	2956 (43)	3453 (50)	3711 (54)	3955 (58)	14,075 (51)
SBP mm Hg (SD)	126 (17)	127 (17)	128 (16)	129 (16)	127 (17)
DBP mm Hg (SD)	76 (10)	76 (10)	77 (10)	77 (10)	77 (10)
eGFR ml/min/1.73m^2^ (SD)	88 (19)	83 (20)	81 (20)	77 (21)	82 (21)
UACR (IQR)	7 [4, 13]	7 [5, 14]	7 [5, 15]	8 [5, 21]	7 [5, 16]
Total cholesterol mg/dL (SD)	189 (37)	189 (38)	191 (40)	199 (44)	192 (40)
LDL cholesterol mg/dL (SD)	109 (32)	115 (34)	118 (35)	115 (38)	114 (35)
HDL cholesterol mg/dL (SD)	67 (17)	54 (12)	47 (11)	39 (9)	52 (16)
Triglycerides mg/dL (SD)	68 [57, 79]	95 [84, 110]	129 [112, 149]	204 [168, 260]	111 [81, 158]
***Measures of Adiposity***					
LAP (IQR)	97 [66, 135]	172 [128, 224]	255 [194, 327]	449 [333, 613]	208 [126, 338]
BMI (SD)	27.2 (5.8)	29.0 (6.2)	30.0 (6.1)	30.8 (5.9)	29.3 (6.1)
WC, cm (SD)	89 (14)	95 (14)	98 (15)	102 (14)	96 (15)

*Abbreviations: CHD* coronary heart disease, *MI* myocardial infarction, *HTN* hypertension, *SBP* systolic blood pressure, *DBP* diastolic blood pressure, *eGFR* estimated glomerular filtration rate, *UACR* urine albumin-to-creatinine ratio, *LDL* low-density lipoprotein cholesterol, *HDL* high-density lipoprotein cholesterol, *LAP* lipid accumulation product, *BMI* body mass index, *WC* waist circumference

### Relationship between measures of obesity and incident CKD

Between the baseline and follow-up visits (median time 6.3 years), there were 1127 cases of incident CKD and 1452 cases of progressive eGFR decline. Each two-fold higher of VAI was associated with 31% higher odds (95% CI 1.24, 1.40) of developing incident CKD in the unadjusted model (Table 3). This association was attenuated but remained statistically significant after adjusting for all the covariates, including baseline eGFR and UACR. Similar associations were seen for LAP where each two-fold higher level associated with 21% higher odds (95% CI 1.13, 1.29) of CKD (Table 3). In categorical analyses, the top quartiles of VAI and LAP were associated with higher odds of incident CKD as compared to the lowest quartile (OR 1.26; 95% CI 1.02, 1.55 and OR 1.51; 95% CI 1.22, 1.87 respectively) in fully adjusted models. BMI and WC were also associated with increased risk of incident CKD in fully adjusted models when compared to the lower quartiles (OR 1.81; 95% CI 1.46, 2.25 and OR 1.64; 95% CI 1.33, 2.01 respectively) as shown in Table 3. We did not find an interaction between VAI or LAP with sex (0.62 and 0.59, respectively).

**Table 3:** Association of measures of adiposity with incident chronic kidney disease (CKD)

	Events/N	Proportion	Unadjusted	Model 1	Model 2	Model 3
			OR (95% CI)	OR (95% CI)	OR (95% CI)	OR (95% CI)
**VAI**						
Continuous (per doubling)	1127/11538	10%	1.31 (1.24, 1.40)	1.41 (1.32, 1.51)	1.23 (1.15, 1.32)	1.12 (1.04, 1.20)
Quartiles						
≤ 2.31	215/3166	7%	*1.00 (ref)*	*1.00 (ref)*	*1.00 (ref)*	*1.00 (ref)*
2.32–3.64	277/3033	9%	1.39 (1.14, 1.68)	1.37 (1.13, 1.67)	1.22 (0.99, 1.48)	1.13 (0.92, 1.38)
3.65–5.91	319/2826	11%	1.82 (1.51, 2.19)	1.94 (1.60, 2.35)	1.61 (1.33, 1.96)	1.40 (1.15, 1.72)
≥ 5.92	316/2513	13%	1.99 (1.65, 2.40)	2.30 (1.89, 2.79)	1.60 (1.31, 1.96)	1.26 (1.02, 1.55)
**LAP**						
Continuous (per doubling)	1127/11538	10%	1.42 (1.33, 1.50)	1.54 (1.44, 1.64)	1.32 (1.23, 1.41)	1.21 (1.13, 1.29)
Quartiles						
≤ 124.89	195/3175	6%	*1.00 (ref)*	*1.00 (ref)*	*1.00 (ref)*	*1.00 (ref)*
124.9–208.07	262/3057	9%	1.40 (1.15, 1.71)	1.33 (1.09, 1.63)	1.16 (0.95, 1.43)	1.12 (0.91, 1.38)
208.08–339.57	325/2794	12%	2.01 (1.67, 2.43)	2.03 (1.67, 2.46)	1.62 (1.32, 1.97)	1.45 (1.18, 1.78)
≥ 339.58	345/2512	14%	2.42 (2.01, 2.93)	2.90 (2.38, 3.52)	1.91 (1.56, 2.35)	1.51 (1.22, 1.87)
**BMI**						
Continuous (per doubling)	1127/11538	10%	2.69 (2.15, 3.37)	4.47 (3.48, 5.74)	2.73 (2.08, 3.56)	2.10 (1.60, 2.76)
Quartiles						
≤ 25.1	191/2849	7%	*1.00 (ref)*	*1.00 (ref)*	*1.00 (ref)*	*1.00 (ref)*
25.2–28.3	273/3096	9%	1.30 (1.06, 1.58)	1.38 (1.13, 1.69)	1.26 (1.03, 1.55)	1.22 (0.99, 1.50)
28.4–32.5	292/2978	10%	1.52 (1.25, 1.84)	1.74 (1.43, 2.13)	1.40 (1.14, 1.72)	1.30 (1.05, 1.60)
> 32.5	371/2615	14%	2.24 (1.86, 2.70)	3.09 (2.53, 3.78)	2.17 (1.75, 2.68)	1.81 (1.46, 2.25)
**Waist Circumference**						
Continuous (per doubling)	1127/11538	10%	4.42 (3.30, 5.92)	7.57 (5.48, 10.47)	3.62 (2.57, 5.10)	2.66 (1.88, 3.77)
Quartiles						
≤ 86.3	256/3508	7%	*1.00 (ref)*	*1.00 (ref)*	*1.00 (ref)*	*1.00 (ref)*
86.4–95.2	215/2710	8%	1.04 (0.86, 1.27)	1.11 (0.91, 1.36)	0.97 (0.79, 1.19)	0.97 (0.78, 1.19)
95.3–105.4	290/2874	10%	1.37 (1.14, 1.64)	1.57 (1.29, 1.91)	1.23 (1.01, 1.50)	1.13 (0.92, 1.38)
> 105.4	366/2446	15%	2.22 (1.87, 2.64)	2.91 (2.41, 3.51)	1.91 (1.57, 2.34)	1.64 (1.33, 2.01)

Model 1: adjusted for age, sex, race

Model 2: Model 1 + prevalent CHD, prevalent stroke, HTN, DM, smoking status

Model 3: Model 2+ eGFR, UACR

*Abbreviations*: *VAI* visceral adiposity index, *LAP* lipid accumulation product, *BMI* body mass index, *CHD* coronary heart disease, *HTN* hypertension, *DM* diabetes mellitus, *eGFR* estimated glomerular filtration rate, *UACR* urine albumin-to-creatinine ratio

### Relationship between measures of obesity and progressive eGFR decline

In the unadjusted model, each two-fold higher level of VAI was associated with 23% higher odds (95% CI 1.17, 1.31) of progressive eGFR decline and this association remained statistically significant after full multivariable adjustment (OR 1.11; 95% CI 1.04, 1.18, Table 4). Similar findings were observed on categorical analyses (Table 4) with the top quartile of VAI being associated with 20% higher odds of eGFR decline as compared to the lowest quartile. Each per two-fold higher and the top quartile of LAP were also associated with odds of progressive eGFR, 18% higher (95% CI 1.11, 1.26) and 36% (95% CI 1.13, 1.63) higher respectfully (Table 4). Each two-fold higher of BMI and WC were also associated with increased risk of progressive eGFR across the models (Table 4).

**Table 4:** Association of measures of adiposity with progressive eGFR decline

	Events/N	Proportion	Unadjusted	Model 1	Model 2	Model 3
			OR (95% CI)	OR (95% CI)	OR (95% CI)	OR (95% CI)
**VAI**						
Continuous (per doubling)	1452/12624	12%	1.23 (1.17, 1.31)	1.31 (1.23, 1.38)	1.14 (1.07, 1.21)	1.11 (1.04, 1.18)
Quartiles						
≤ 2.31	306/3304	9%	*1.00 (ref)*	*1.00 (ref)*	*1.00 (ref)*	*1.00 (ref)*
2.32–3.64	355/3275	11%	1.18 (1.00, 1.39)	1.16 (0.98, 1.38)	1.02 (0.86, 1.22)	1.02 (0.86, 1.22)
3.65–5.91	378/3143	12%	1.37 (1.17, 1.62)	1.43 (1.22, 1.69)	1.16 (0.97, 1.37)	1.14 (0.95, 1.35)
≥ 5.92	413/2902	14%	1.62 (1.37, 1.90)	1.83 (1.55, 2.16)	1.27 (1.07, 1.51)	1.20 (1.00, 1.44)
**LAP**						
Continuous (per doubling)	1452/12624	12%	1.35 (1.28, 1.43)	1.42 (1.34, 1.50)	1.21 (1.14, 1.29)	1.18 (1.11, 1.26)
Quartiles						
≤ 124.89	264/3311	8%	*1.00 (ref)*	*1.00 (ref)*	*1.00 (ref)*	*1.00 (ref)*
124.9–208.07	340/3305	10%	1.29 (1.08, 1.53)	1.20 (1.01, 1.43)	1.05 (0.88, 1.25)	1.06 (0.88, 1.26)
208.08–339.57	406/3107	13%	1.71 (1.44, 2.02)	1.65 (1.39, 1.96)	1.30 (1.09, 1.54)	1.30 (1.09, 1.56)
≥ 339.58	442/2901	15%	2.06 (1.75, 2.44)	2.25 (1.90, 2.66)	1.45 (1.22, 1.74)	1.36 (1.13, 1.63)
**BMI**						
Continuous (per doubling)	1452/12624	12%	2.83 (2.32, 3.45)	3.19 (2.58, 3.95)	1.89 (1.50, 2.37)	1.79 (1.42, 2.25)
Quartiles						
≤ 25.1	238/3042	8%	*1.00 (ref)*	*1.00 (ref)*	*1.00 (ref)*	*1.00 (ref)*
25.2–28.3	337/3338	10%	1.29 (1.08, 1.55)	1.29 (1.08, 1.55)	1.18 (0.98, 1.42)	1.22 (1.01, 1.47)
28.4–32.5	389/3271	12%	1.61 (1.36, 1.92)	1.64 (1.37, 1.95)	1.32 (1.1.0, 1.59)	1.33 (1.10, 1.60)
> 32.5	488/2973	16%	2.29 (1.93, 2.71)	2.44 (2.05, 2.91)	1.67 (1.38, 2.01)	1.63 (1.34, 1.97)
**Waist Circumference**						
Continuous (per doubling)	1452/12624	12%	4.22 (3.26, 5.48)	5.16 (3.91, 6.81)	2.42 (1.80, 3.25)	2.21 (1.64, 2.99)
Quartiles						
≤ 86.3	307/3739	8%	*1.00 (ref)*	*1.00 (ref)*	*1.00 (ref)*	*1.00 (ref)*
86.4–95.2	298/2936	10%	1.20 (1.01, 1.43)	1.23 (1.03, 1.48)	1.07 (0.89, 1.29)	1.12 (0.93, 1.34)
95.3–105.4	375/3172	12%	1.44 (1.23, 1.70)	1.53 (1.29, 1.81)	1.20 (1.01, 1.43)	1.20 (1.00, 1.44)
> 105.4	472/2777	17%	2.27 (1.94, 2.66)	2.51 (2.13, 2.96)	1.63 (1.37, 1.95)	1.58 (1.32, 1.89)

Model 1: adjusted for age, sex, race

Model 2: Model 1 + prevalent CHD, prevalent stroke, HTN, DM, smoking status

Model 3: Model 2+ eGFR, UACR

*Abbreviations: VAI* visceral adiposity index, *LAP* lipid accumulation product, *BMI* body mass index, *CHD* coronary heart disease, *HTN* hypertension, *DM* diabetes mellitus, *eGFR* estimated glomerular filtration rate, *UACR* urine albumin-to-creatinine ratio

### Relationship between measures of obesity and incident kidney failure

There were 353 cases of incident kidney failure over 10 years of follow up. Incidence rates of kidney failure were higher with ascending quartiles of VAI (0.11%/year in Q1 to 0.24%/year in Q4) and LAP (0.08%/year in Q1 to 0.29%/year in Q4). Each two-fold higher of VAI was associated with a 38% higher risk (95% CI 1.25, 1.53) of incident kidney failure in unadjusted models (Table 5). This finding remained statistically significant despite adjusting for age, sex, race, prevalent CHD, stroke, hypertension, diabetes mellitus, and smoking status (HR:1.33 and 95% CI 1.19, 1.49). However, addition of eGFR and UACR to the model attenuated these associations almost completely. Similarly, although, each two-fold higher of LAP was associated with greater risk of incident kidney failure in unadjusted, and demographic adjusted models, the inclusion of prevalent co-morbidities, eGFR and UACR rendered this association no longer statistically significant (Table 5) on the continuous per two-fold scale. Similar findings were also seen in categorical analyses where the top quartile of VAI (HR: 1.94; 95% CI 1.37, 2.76) and LAP (HR: 2.03; 95% CI 1.39, 2.97) were associated with risk of incident kidney failure only before but not after adjustment for eGFR and UACR. In comparison, BMI and WC were also associated with risk of kidney failure in an unadjusted and demographic adjusted models. However, further adjustment for eGFR and UACR resulted in a statistically significant inverse relationship between higher BMI and WC and risk of incident kidney failure (Table 5). Supplemental tables 1 and 2 shows the association of VAI and LAP, respectively, and the rest of the covariates with kidney failure before and after adding albuminuria and baseline eGFR to the analysis.

**Table 5:** Association of measures of adiposity with incident kidney failure

	Events/N	Incidence rate (%/yr)	Unadjusted	Model 1	Model 2	Model 3
			HR (95% CI)	HR (95% CI)	HR (95% CI)	HR (95% CI)
**VAI**						
Continuous (per doubling)	353/27550	0.17	1.38 (1.25, 1.53)	1.63 (1.47, 1.81)	1.33 (1.19, 1.49)	0.93 (0.82, 1.04)
Quartiles						
≤ 2.31	56/6873	0.11	*1.00 (ref)*	*1.00 (ref)*	*1.00 (ref)*	1.00 (ref)
2.32–3.64	64/6915	0.12	1.17 (0.80, 1.70)	1.27 (0.87, 1.85)	1.06 (0.73, 1.55)	0.75 (0.51, 1.10)
3.65–5.91	111/6886	0.22	2.01 (1.43, 2.82)	2.44 (1.74, 3.43)	1.71 (1.21, 2.41)	1.02 (0.72, 1.44)
≥ 5.92	122/6876	0.24	2.20 (1.57, 3.07)	3.42 (2.43, 4.80)	1.94 (1.37, 2.76)	0.72 (0.50, 1.03)
**LAP**						
Continuous (per doubling)	353/27550	0.17	1.48 (1.33, 1.64)	1.67 (1.49, 1.86)	1.26 (1.12, 1.42)	0.89 (0.81, 0.99)
Quartiles						
≤ 124.89	39/6838	0.08	*1.00 (ref)*	*1.00 (ref)*	*1.00 (ref)*	1.00 (ref)
124.9–208.07	74/6943	0.14	1.74 (1.17, 2.61)	1.63 (1.09, 2.44)	1.25 (0.83, 1.87)	0.99 (0.66, 1.49)
208.08–339.57	96/6933	0.19	2.32 (1.58, 3.40)	2.32 (1.58, 3.40)	1.49 (1.01, 2.20)	1.00 (0.67, 1.49)
≥ 339.58	144/6836	0.29	3.48 (2.42, 5.02)	4.26 (2.95, 6.14)	2.03 (1.39, 2.97)	0.82 (0.56, 1.20)
**BMI**						
Continuous (per doubling)	353/27550	0.17	2.50 (1.72, 3.63)	2.08 (1.40, 3.10)	0.80 (0.52, 1.23)	0.55 (0.37, 0.81)
Quartiles						
≤ 25.1	60/6866	0.12	1.00 (ref)	1.00 (ref)	1.00 (ref)	1.00 (ref)
25.2–28.3	80/6968	0.15	1.22 (0.86, 1.72)	1.11 (0.79, 1.57)	0.86 (0.61, 1.22)	0.95 (0.67, 1.35)
28.4–32.5	87/6964	0.17	1.32 (0.94, 1.85)	1.13 (0.80, 1.60)	0.70 (0.50, 1.00)	0.58 (0.410, 0.84)
> 32.5	126/6792	0.25	1.95 (1.42, 2.69)	1.64 (1.18, 2.28)	0.76 (0.54, 1.08)	0.58 (0.40, 0.82)
**Waist Circumference**						
Continuous (per doubling)	353/27550	0.17	5.57 (3.46, 8.97)	4.27 (2.56, 7.13)	1.06 (0.62, 1.82)	0.43 (0.26, 0.70)
Quartiles						
≤ 86.3	56/7794	0.10	1.00 (ref)	1.00 (ref)	1.00 (ref)	1.00 (ref)
86.4–95.2	63/6204	0.13	1.29 (0.89, 1.87)	1.08 (0.74, 1.57)	0.82 (0.57, 1.20)	0.84 (0.58, 1.22)
95.3–105.4	95/6977	0.18	1.73 (1.23, 2.43)	1.43 (1.01, 2.03)	0.89 (0.63, 1.27)	0.74 (0.52, 1.05)
> 105.4	139/6615	0.29	2.73 (1.98, 3.76)	2.19 (1.58, 3.04)	0.99 (0.70, 1.39)	0.55 (0.39, 0.78)

Model 1: adjusted for age, sex, race

Model 2: Model 1 + prevalent CHD, prevalent stroke, HTN, DM, smoking status

Model 3: Model 2+ eGFR, UACR

*Abbreviations: VAI* visceral adiposity index, *LAP* lipid accumulation product, *BMI* body mass index, *CHD* coronary heart disease, *HTN* hypertension, *DM* diabetes mellitus, *eGFR* estimated glomerular filtration rate, *UACR* urine albumin-to-creatinine ratio

In sensitivity analysis stratifying by eGFR categories, we found statistically non-significant higher risk of kidney failure per two-fold VAI, LAP and kidney failure after multivariable adjustment in persons with eGFR < 45 ml/min/1.73m^2^. The association of BMI and WC with kidney failure in these GFR categories was statistically non-significant, the point estimates were in the opposite direction from VAI and LAP. We found similar results when treating death as a competing risk (Supplemental Table 3). Among those with eGFR < 45 ml/min/1.73m^2^, higher LAP, BMI and WC were all associated with lower risk of kidney failure after multivariable adjustment (Supplemental Table 4).

Wald test statistics only demonstrated a significant contribution by WC when compared to VAI, LAP, and BMI (0–10%) in the prediction of kidney failure (Supplemental Fig. 1).

Lastly, we did not find a significant association between HDL with any of the outcomes whereas we found weak associations between triglycerides and incident CKD and progressive eGFR decline (Supplemental Table 5).

### Relationship between measures of obesity and incident albuminuria

Between the baseline and follow-up visits, there were 1397 cases of incident albuminuria. Each two-fold higher of VAI was associated with 21% higher odds (95% CI 1.14, 1.28) of developing incident albuminuria in the unadjusted model (Supplemental Table 6). This association was attenuated and no longer statistically significant after adjusting for all the covariates, including baseline eGFR and baseline UACR (OR 1.04; 95% CI 0.97, 1.11). Similarly, in the unadjusted model, a two-fold higher level of LAP was associated with 18% higher odds (95% CI 1.12, 1.24) of incident albuminuria, but this association was no longer significant after adjusting for all covariates (OR 0.98, 95% CI 0.93, 1.05) as shown in Supplemental Table 6.

## Discussion

This study, conducted in a large population of adults at risk for kidney and cardiovascular disease showed that the VAI and LAP, newer anthropometric measures of visceral adiposity, were associated with incident CKD and progressive decline in kidney function, after adjusting for multiple confounders including eGFR and albuminuria. These associations were similar in strength and direction to those of BMI and WC. In demographic adjusted models, VAI and LAP were associated with risk of incident kidney failure but these associations were not independent of the baseline level of eGFR and albuminuria.

Prior cross-sectional analyses among Chinese participants demonstrated a 2.32 fold higher prevalence of reduced kidney function defined as eGFR < 60 mL/min per 1.73 m^2^ among persons in the highest quartile of LAP compared to the lowest quartile [29]. Another cross-sectional study in a rural Chinese population (11,192 participants total) showed both VAI and LAP were associated with over four fold higher odds of CKD in women [26]. This study differed from our study since their fully adjusted model did not account for diabetes mellitus and proteinuria. Additionally, due to the cross-sectional nature of these studies, they were not able to adjust for baseline kidney function decline. In another study, after adjusting for confounders (not including baseline eGFR) the association between VAI and prevalent CKD was not statistically significant [30]. Our study adds to the existing literature, demonstrating the longitudinal associations of these visceral adiposity measures with future kidney function decline after adjusting for known confounders, and comparing strengths to those of the more established adiposity markers of BMI and WC.

In two population based cohorts from the Tehran Lipid and Glucose study and the third National Health and Nutrition Examination Survey (NHANES III), LAP has been associated with a higher risk of cardiovascular disease when compared to BMI [22, 31, 32]. LAP has also been shown to be associated with risk of all-cause mortality compared to BMI in nondiabetic patients at high risk for cardiovascular diseases [33], as well as congestive heart failure mortality and all-cause mortality in postmenopausal women [34]. Similarly, VAI has been associated with a higher risk of cardiovascular disease [23]. This may be due to VAI including physical and metabolic parameters and indirectly reflecting altered production of adipocytokines, increased lipolysis, and plasma free fatty acids, which are not captured by traditional markers such as BMI, WC, TGs, and HDL, separately [23].

In our study, we found a dose-dependent relationship between higher levels of all the obesity markers evaluated and incident kidney failure until adjustment for eGFR and albuminuria. To obtain a better understanding of this phenomenon, we performed sensitivity analyses accounting for the competing risk of death and also performed stratified analysis according to baseline eGFR categories (> 60, 45–59, and < 45 ml/min/1.73m^2^). These sensitivity analyses did not alter our results. Interestingly, the inverse association between BMI and incident kidney failure has also been shown in prior studies in REGARDS [15]. After adjusting for WC and obesity-associated co-morbidities and eGFR, BMI categories ≥35 kg/m^2^ were associated with significantly lower kidney failure risk [15]. Other studies have also failed to find an association between higher BMI and incident CKD [7, 35–37]. In a cohort of more than 300 patients with stages 3–5 CKD, Lin et al. found that a BMI ≥ to 30 or a body fat percentage > 25% in men and > 35% in women did not confer an increased risk of kidney failure [38]. Wang et al. also found that BMI was not a risk factor for kidney failure in either males or females with CKD and mild obesity (BMI 30–34.9 kg/m^2^) was associated with lower risk for death without RRT in males (HR: 0.60 95% CI: 0.40–0.90, *p* = 0.013) [35]. Mechanisms responsible for this inverse association are uncertain.

### Strengths and limitations

Our study has limitations. First, there was a relatively short follow up time to adequately assess development of kidney failure. Second, although studies have shown that VAI and LAP are correlated with visceral adiposity our study lacks direct measures of visceral adiposity such as CT and MRI measures of adiposity. Strengths of this study include a large cohort of adults from different racial backgrounds with CKD and a longitudinal approach to evaluating the association of LAP and VAI with clinical endpoints.

In conclusion, VAI and LAP, newer visceral adiposity measures, are associated with incident CKD and eGFR decline after adjusting for multiple potential confounders including eGFR and albuminuria; however, BMI and WC, traditional measures, were more strongly associated with these outcomes. Future studies should compare measures of visceral adiposity by direct and indirect methods to evaluate if the risk of adverse kidney events are different and whether better noninvasive markers of visceral adiposity are needed to differentiate the subgroup of patients who are at higher risk of developing worse outcomes.

## Supplementary Information

**Additional file 1: Supplemental Table 1.** Association of VAI with incident kidney failure. **Supplemental Table 2.** Association of LAP with incident kidney failure. **Supplemental Table 3.** Association of measures of adiposity with incident kidney failure in a competing risk of death analysis. **Supplemental Table 4.** Association of measures of adiposity with incident kidney failure stratified by baseline CKD stages. **Supplemental Table 5.** Association of triglycerides and HDL with incident CKD, progressive eGFR decline, and incident kidney failure. **Supplemental Table 6.** Association of measures of adiposity with incident albuminuria. **Supplemental Figure 1.** Global Wald Chi-Square Score of Relative Importance of Measures of Adiposity to Incident Kidney Failure. Abbreviations: VAI, visceral adiposity index; BMI, body mass index; LAP, lipid accumulation product.

## Data Availability

All data generated or analyzed during this study are included in this published article and its supplementary information files.
