# Supplementary material for: Lipid accumulation product, visceral adiposity index and risk of chronic kidney disease
Source: BMC Nephrol. 2022 Dec 15;23:401. doi: 10.1186/s12882-022-03026-9 (PMC9753382; doi:10.1186/s12882-022-03026-9)
Supplement: Supplementary file 1 — Additional file 1: Supplemental Table 1. Association of VAI with incident kidney failure. Supplemental Table 2. Association of LAP with incident kidney failure. Supplemental Table 3. Association of measures of adiposity with incident kidney failure in a competing risk of death analysis. Supplemental Table 4. Association of measures of adiposity with incident kidney failure stratified by baseline CKD stages. Supplemental Table 5. Association of triglycerides and HDL with incident CKD, progressive eGFR decline, and incident kidney failure. Supplemental Table 6. Association of measures of adiposity with incident albuminuria. Supplemental Figure 1. Global Wald Chi-Square Score of Relative Importance of Measures of Adiposity to Incident Kidney Failure. Abbreviations: VAI, visceral adiposity index; BMI, body mass index; LAP, lipid accumulation product. [file 12882_2022_3026_MOESM1_ESM.docx]

| **Supplemental Table 1. Association of VAI with incident kidney failure**   \|  \| Events/N \| Incidence rate (%/yr) \| HR  (95% CI) \| \| \| \| --- \| --- \| --- \| --- \| --- \| --- \| \| **VAI**  Continuous  (per two-fold) \| 353/27550 \| 0.17 \| 1.33 (1.19, 1.49) \| 1.16 (1.04, 1.30) \| 0.93 (0.82, 1.04) \| \| Age \|  \|  \| 1.02 (1.01, 1.04) \| 1.02 (1.01, 1.03) \| 0.97 (0.96, 0.99) \| \| Sex \|  \|  \| 0.74 (0.59, 0.94) \| 0.92 (0.73, 1.17) \| 0.79 (0.63, 1.01) \| \| Race \|  \|  \| 1.91 (1.67, 2.19) \| 1.63 (1.42, 1.86) \| 1.60 (1.40, 1.84) \| \| Prevalent CHD \|  \|  \| 1.61 (1.26, 2.05) \| 1.44 (1.13, 1.83) \| 0.98 (0.77, 1.84) \| \| Prevalent stroke \|  \|  \| 1.63 (1.20, 2.24) \| 1.16 (0.85, 1.59) \| 1.04 (0.76, 1.42) \| \| HTN \|  \|  \| 3.74 (2.52, 5.56) \| 2.42 (1.62, 3.61) \| 1.57 (1.04, 2.36) \| \| DM \|  \|  \| 3.86 (3.04, 4.89) \| 1.74 (1.36, 2.24) \| 1.53 (1.18, 1.99) \| \| Smoking status \|  \|  \| 1.16 (0.91, 1.50) \| 1.03 (0.80, 1.33) \| 1.35 (1.04, 1.74) \| \| UACR \|  \|  \|  \| 1.75 (1.69, 1.83) \| 1.43 (1.36, 1.49) \| \| eGFR \|  \|  \|  \|  \| 0.94 (0.93, 0.94) \|   Abbreviations: Abbreviations: VAI, visceral adiposity index; CHD, coronary heart disease; HTN, hypertension; DM, diabetes mellitus; eGFR, estimated glomerular filtration rate; UACR, urine albumin-to-creatinine ratio  **Supplemental Table 2. Association of LAP with incident kidney failure**   \|  \| Events/N \| Incidence rate (%/yr) \| HR  (95% CI) \| \| \| \| --- \| --- \| --- \| --- \| --- \| --- \| \| **LAP**  Continuous  (per two-fold) \| 353/27550 \| 0.17 \| 1.26 (1.12, 1.42) \| 1.06 (0.95, 1.18) \| 0.89 (0.81, 0.99) \| \| Age \|  \|  \| 1.03 (1.01, 1.04) \| 1.02 (1.01, 1.03) \| 0.97 (0.96, 0.99) \| \| Sex \|  \|  \| 0.77 (0.61, 0.97) \| 0.95 (0.75, 1.20) \| 0.79 (0.62, 1.00) \| \| Race \|  \|  \| 1.84 (1.61, 2.10) \| 1.56 (1.37, 1.79) \| 1.60 (1.40, 1.83) \| \| Prevalent CHD \|  \|  \| 1.62 (1.27, 2.07) \| 1.45 (1.14, 1.85) \| 0.98 (0.77, 1.25) \| \| Prevalent stroke \|  \|  \| 1.68 (1.23, 2.29) \| 1.18 (0.86, 1.61) \| 1.03 (0.75, 1.40) \| \| HTN \|  \|  \| 3.65 (2.45, 5.43) \| 2.44 (1.64, 3.65) \| 1.61 (1.07, 2.42) \| \| DM \|  \|  \| 3.83 (3.01, 4.88) \| 1.80 (1.40, 2.31) \| 1.57 (1.21, 2.04) \| \| Smoking status \|  \|  \| 1.17 (0.91, 1.50) \| 1.03 (0.80, 1.33) \| 1.34 (1.04, 1.74) \| \| UACR \|  \|  \|  \| 1.76 (1.69, 1.83) \| 1.43 (1.37, 1.50) \| \| eGFR \|  \|  \|  \|  \| 0.94 (0.93, 0.94) \|   Abbreviations: Abbreviations: LAP, lipid accumulation product; CHD, coronary heart disease; HTN, hypertension; DM, diabetes mellitus; eGFR, estimated glomerular filtration rate; UACR, urine albumin-to-creatinine ratio  **Supplemental Table 3. Association of measures of adiposity with incident kidney failure in a competing risk of death analysis** | | | | | | |
| --- | --- | --- | --- | --- | --- | --- | --- | --- | --- | --- | --- | --- | --- | --- | --- | --- | --- | --- | --- | --- | --- | --- | --- | --- | --- | --- | --- | --- | --- | --- | --- | --- | --- | --- | --- | --- | --- | --- | --- | --- | --- | --- | --- | --- | --- | --- | --- | --- | --- | --- | --- | --- | --- | --- | --- | --- | --- | --- | --- | --- | --- | --- | --- | --- | --- | --- | --- | --- | --- | --- | --- | --- | --- | --- | --- | --- | --- | --- | --- | --- | --- | --- | --- | --- | --- | --- | --- | --- | --- | --- | --- | --- | --- | --- | --- | --- | --- | --- | --- | --- | --- | --- | --- | --- | --- | --- | --- | --- | --- | --- | --- | --- | --- | --- | --- | --- | --- | --- | --- | --- | --- | --- | --- | --- | --- | --- | --- | --- | --- | --- | --- | --- | --- | --- | --- | --- | --- | --- | --- | --- | --- | --- | --- | --- | --- | --- | --- | --- | --- | --- |
|  |  |  |  |  |  |  |
|  |  | **Incidence rate (%/yr)** |  |  |  |  |
|  | **Events/N** |  | **Unadjusted** | **Model 1** | **Model 2** | **Model 3** |
|  |  |  | HR (95% CI) | HR (95% CI) | HR (95% CI) | HR (95% CI) |
| **VAI** |  |  |  |  |  |  |
| Continuous | comp risk death |  | 1.37 (1.23, 1.51) | 1.61 (1.45, 1.79) | 1.32 (1.18, 1.48) | 0.95 (.085, 1.06) |
| (per doubling) | 353/27550 | 0.17 | 1.38 (1.25, 1.53) | 1.63 (1.47, 1.81) | 1.33 (1.19, 1.49) | 0.93 (0.82, 1.04) |
| **LAP** |  |  |  |  |  |  |
| Continuous | comp risk death |  | 1.45 (1.31, 1.61) | 1.63 (1.46, 1.82) | 1.25 (1.11, 1.40) | 0.92 (0.83, 1.2) |
| (per doubling) | 353/27550 | 0.17 | 1.48 (1.33, 1.64) | 1.67 (1.49, 1.86) | 1.26 (1.12, 1.42) | 0.89 (0.81, 0.99) |
| **BMI** |  |  |  |  |  |  |
| Continuous | comp risk death |  | 2.58 (1.79, 3.72) | 2.7 (1.40, 3.06) | 0.85 (0.56, 1.28) | 0.63 (0.43, 0.92) |
| (per doubling) | 353/27550 | 0.17 | 2.50 (1.72, 3.63) | 2.08 (1.40, 3.10) | 0.80 (0.52, 1.23) | 0.55 (0.37, 0.81) |
| **Waist**  **Circumference** | |  |  |  |  |  |
| Continuous | comp risk death |  | 5.16 (3.20, 8.33) | 3.96 (2.38, 6.58) | 1.06 (0.63, 1.79) | 0.45 (0.28, 0.74) |
| (per doubling) | 353/27550 | 0.17 | 5.57 (3.46, 8.97) | 4.27 (2.56, 7.13) | 1.06 (0.62, 1.82) | 0.43 (0.26, 0.70) |
| Abbreviations: VAI, visceral adiposity index; LAP, lipid accumulation product; BMI, body mass index; CAD, coronary artery disease; HTN, hypertension; DM, diabetes mellitus; eGFR, estimated glomerular filtration rate; UACR, urine albumin-to-creatinine ratio | | | | | | |
| Model 1: adjusted for age, sex, race | | | |  |  |  |
| Model 2: Model 1 + prevalent CAD, prevalent stroke, HTN, DM, smoking status | | | | | |  |
| Model 3: Model 2+ eGFR, UACR | | | | |  |  |

| **Supplemental Table 4. Association of measures of adiposity with incident kidney failure stratified by baseline CKD stages** | | | | | | | |
| --- | --- | --- | --- | --- | --- | --- | --- |
|  | **N** | **# Kidney failure events** | **rate (%/yr)** | **VAI**  **(per doubling)*** | **LAP (per doubling)*** | **BMI**  **(per doubling)*** | **Waist Circumference (per doubling)*** |
|  |  |  |  | HR (95% CI) | HR (95% CI) | HR (95% CI) | HR (95% CI) |
| **Baseline eGFR** |  |  |  |  |  |  |  |
| ≥ 60 | 23564 | 86 | 0.05 | 1.18 (0.95, 1.48) | 1.13 (0.91, 1.40) | 0.96 (0.42, 2.20) | 0.82 (0.30, 2.23) |
| 45 -- 59 | 2561 | 52 | 0.29 | 1.10 (0.82, 1.48) | 1.08 (0.80, 1.40) | 0.77 (0.31, 1.96) | 0.75 (0.21, 2.60) |
| < 45 | 1465 | 215 | 2.57 | 0.90 (0.77, 1.05) | 0.86 (0.75, 0.98) | 0.57 (0.34, 0.93) | 0.43 (0.21, 0.88) |
| Abbreviations: CKD, chronic kidney disease; VAI, visceral adiposity index; LAP, lipid accumulation product; BMI, body mass index; eGFR, estimated glomerular filtration rate; CHD, coronary heart disease; HTN, hypertension; DM, diabetes mellitus; UACR, urine albumin-to-creatinine ratio  * adjusted for age, sex, race, prevalent CHD, prevalent stroke, HTN, DM, smoking status, and UACR | | | | | | | |

| **Supplemental Table 5. Association of triglycerides and HDL with incident CKD, progressive eGFR decline, and incident kidney failure** | | |
| --- | --- | --- |
|  | **Triglycerides (per doubling) *** | **HDL (per doubling) *** |
|  | HR/OR (95% CI) | HR/OR (95% CI) |
| **Outcome** |  |  |
| Incident chronic kidney disease | 1.18 (1.07, 1.30) | 0.91 (0.77, 1.09) |
| Progressive eGFR decline | 1.17 (1.07, 1.27) | 0.94 (0.81, 1.10) |
| Incident kidney failure | 0.98 (0.84, 1.14) | 0.78 (0.59, 1.04) |
| Abbreviations: HDL, high-density lipoprotein cholesterol; eGFR, estimated glomerular filtration rate; CKD, chronic kidney disease; CHD, coronary heart disease; HTN, hypertension; DM, diabetes mellitus; UACR, urine albumin-to-creatinine ratio  * adjusted for age, sex, race, prevalent CHD, prevalent stroke, HTN, DM, smoking status, UACR, & eGFR | | |

**Supplemental Table 6. Association of measures of adiposity with incident albuminuria**

|  | **Events/N** | **Proportion** | **Unadjusted** | **Model 1** | **Model 2** | **Model 3** |
| --- | --- | --- | --- | --- | --- | --- |
|  |  |  | OR (95% CI) | OR (95% CI) | OR (95% CI) | OR (95% CI) |
| **VAI** |  |  |  |  |  |  |
| Continuous (per doubling) | 1397/10893 | 13% | 1.21 (1.14, 1.28) | 1.25 (1.18, 1.33) | 1.11 (1.04, 1.18) | 1.04 (0.97, 1.11) |
| Quartiles |  |  |  |  |  |  |
| ≤ 2.31 | 317/2916 | 11% | *1.00 (ref)* | *1.00 (ref)* | *1.00 (ref)* | *1.00 (ref)* |
| 2.32 - 3.64 | 324/2887 | 11% | 1.05 (0.89, 1.24) | 1.05 (0.89, 1.24) | 0.95 (0.80, 1.13) | 0.90 (0.75, 1.07) |
| 3.65 - 5.91 | 374/2707 | 14% | 1.33 (1.13, 1.56) | 1.36 (1.15, 1.60) | 1.13 (0.96, 1.34) | 1.03 (0.87, 1.23) |
| ≥ 5.92 | 382/2383 | 16% | 1.57 (1.33, 1.84) | 1.71 (1.45, 2.02) | 1.26 (1.06, 1.50) | 1.07 (0.89, 1.29) |
| **LAP** |  |  |  |  |  |  |
| Continuous (per doubling) | 1397/10893 | 13% | 1.18 (1.12, 1.24) | 1.20 (1.14, 1.27) | 1.05 (0.99, 1.11) | 0.98 (0.93, 1.05) |
| Quartiles |  |  |  |  |  |  |
| ≤ 124.89 | 310/2968 | 10% | *1.00 (ref)* | *1.00 (ref)* | *1.00 (ref)* | *1.00 (ref)* |
| 124.9 - 208.07 | 339/2904 | 12% | 1.12 (0.94, 1.32) | 1.05 (0.89, 1.24) | 0.94 (0.79, 1.11) | 0.91 (0.76, 1.08) |
| 208.08 - 339.57 | 366/2703 | 14% | 1.35 (1.15, 1.59) | 1.30 (1.10, 1.53) | 1.06 (0.89, 1.25) | 0.98 (0.82, 1.17) |
| ≥ 339.58 | 382/2318 | 17% | 1.68 (1.42, 1.97) | 1.78 (1.51, 2.11) | 1.24 (1.04, 1.48) | 1.03 (0.85, 1.24) |
| **BMI** |  |  |  |  |  |  |
| Continuous (per doubling) | 1397/10893 | 13% | 1.45 (1.18, 1.78) | 1.72 (1.38, 2.14) | 1.07 (0.85, 1.36) | 0.93 (0.73, 1.19) |
| Quartiles |  |  |  |  |  |  |
| ≤ 25.1 | 316/2696 | 12% | *1.00 (ref)* | *1.00 (ref)* | *1.00 (ref)* | *1.00 (ref)* |
| 25.2 - 28.3 | 327/2963 | 11% | 0.92 (0.78, 1.09) | 0.91 (0.77, 1.08) | 0.84 (0.71, 0.99) | 0.82 (0.69, 0.99) |
| 28.4 - 32.5 | 387/2825 | 14% | 1.19 (1.02, 1.40) | 1.22 (1.03, 1.43) | 1.02 (0.86, 1.21) | 0.98 (0.82, 1.17) |
| > 32.5 | 367/2409 | 15% | 1.33 (1.13, 1.56) | 1.50 (1.26, 1.78) | 1.07 (0.89, 1.29) | 0.98 (0.81, 1.19) |
| **Waist Circumference** |  |  |  |  |  |  |
| Continuous (per doubling) | 1397/10893 | 13% | 2.11 (1.62, 2.75) | 2.19 (1.65, 2.91) | 1.10 (0.81, 1.49) | 0.86 (0.63, 1.18) |
| Quartiles |  |  |  |  |  |  |
| ≤ 86.3 | 363/3357 | 11% | *1.00 (ref)* | *1.00 (ref)* | *1.00 (ref)* | *1.00 (ref)* |
| 86.4 - 95.2 | 299/2575 | 12% | 1.08 (0.92, 1.28) | 1.04 (0.88, 1.24) | 0.93 (0.78, 1.11) | 0.95 (0.79, 1.14) |
| 95.3 - 105.4 | 353/2725 | 13% | 1.24 (1.05, 1.45) | 1.20 (1.01, 1.41) | 0.99 (0.83, 1.18) | 0.93 (0.78, 1.12) |
| > 105.4 | 382/2236 | 17% | 1.69 (1.45, 1.98) | 1.74 (1.48, 2.06) | 1.20 (1.01, 1.44) | 1.09 (0.91, 1.32) |

| Abbreviations: VAI, visceral adiposity index; LAP, lipid accumulation product; BMI, body mass index; CHD, coronary heart disease; HTN, hypertension; DM, diabetes mellitus; eGFR, estimated glomerular filtration rate; UACR, urine albumin-to-creatinine ratio  Model 1: adjusted for age, sex, race |
| --- |
| Model 2: Model 1 + prevalent CHD, prevalent stroke, HTN, DM, smoking status |
| Model 3: Model 2+ eGFR + baseline UACR |


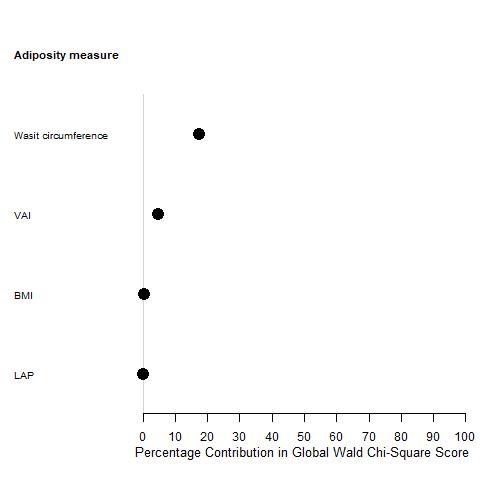


**Supplemental Figure 1. Global Wald Chi-Square Score of Relative Importance of Measures of Adiposity to Incident Kidney Failure**

Abbreviations: VAI, visceral adiposity index; BMI, body mass index; LAP, lipid accumulation product
